# Poor London!

**Published:** 1892-12-10

**Authors:** 


### PASSING TOPICS.

### POOR LONDON!

The "Edinburgh Publisher," as the newspapers have designated the gentleman who bore the very English name of Thomas Nelson, and who carried on business in London as well as in the Scottish capital, has left behind him a splendid fortune, and, unlike too many other rich men, he has devoted a very considerable portion of his wealth to works of public charity. It is, however, worthy of remark that, when we come to glance at the long list of goodly bequests to benevolent purposes, we find this posthumous generosity is wholly and exclusively bestowed upon Edinburgh and Scottish institutions.

Of course, a testator has the right to do what he will with his own; but there are different ways of making use of this liberty, and to the observer, some appear more reasonable than others. At any rate, without calling into question the testator's perfect freedom to do as he pleases, a dedication of property to public purposes, carried out upon lines so curiously partial, invites comment, and not less because it is by no means a singular though undoubtedly a conspicuous illustration of a disposition to overlook the wants of metropolitan institutions.

It will be remembered that blunt old Samuel Johnson, upon hearing the exclamation by a pessimist of his day, "Poor old England is lost," observed that, to his mind, a still more deplorable fact was that the Scotch had found her. But it is not Scotchmen alone who need be invidiously described as apt to give an undue preference to local claims when the time comes for apportioning the sheaves gleaned on metro politan fields. Evidence is continually forthcoming tha men who have made good use of the metropolis for purposes of business or pleasure are somewhat slow to recognize the claims of its poor, and with scarcely concealed vanity they prefer that their benefactions shall be prominently displayed as curiosities elsewhere rather than be made unobtrusively useful in the bye-ways of teeming London.

By all means let the amiable disposition to look back affectionately and gratefully from afar upon our native villages be honoured and encouraged, but something is also due to the great city which has given scope for our energies and compared with which even distinguished and historic Edinburgh offers but a narrow field for vaulting ambition. London, no doubt, owes much of its robust strength to the constant recruiting of the ranks of its inhabitants by provincial and even foreign aspirants, and we do not begrudge intelligent and industrious new-comers what share they legitimately appropriate of its grandeur and riches, but it ought not to be forgotten that these advantages are sometimes obtained at the expense of native competitors, and we surely do not need to add that if London's wealth is great, her poverty is prodigious. We have no desire that local needs should obtain less than just recognition, but we submit that, without entering upon odious comparisons, the metropolis has a claim upon its adopted, no less than upon its begotten children.

Agricola.

